# Visceral-to-subcutaneous fat ratio and risk of gallstones in Korean men: an observational study of 4,914 cases

**DOI:** 10.3389/fmed.2025.1720552

**Published:** 2025-12-08

**Authors:** Hoonsub So, Young-Jee Jeon, Doo-Ho Lim

**Affiliations:** 1 Department of Internal Medicine, Ulsan University Hospital, University of Ulsan College of Medicine, Ulsan, Republic of Korea; 2 Department of Family Medicine, Ulsan University Hospital, University of Ulsan College of Medicine, Ulsan, Republic of Korea

**Keywords:** visceral-to-subcutaneous fat ratio, gallstone, computed tomography, abdominal fat distribution, men

## Abstract

**Introduction:**

Gallstones are a prevalent gastrointestinal disorder influenced by metabolic factors and obesity. Visceral adiposity is metabolically active and proinflammatory, yet the role of the relative distribution of visceral and subcutaneous fat, quantified as the visceral-to-subcutaneous fat ratio (VSR), in gallstone formation remains unclear. This study aimed to investigate the association between VSR, measured by computed tomography (CT), and the prevalence of gallstones in Korean men.

**Methods:**

This retrospective cross-sectional study included 4,914 Korean men who underwent both abdominopelvic CT and ultrasonography as part of routine health examinations. Visceral and subcutaneous fat areas were quantified at the L3 vertebral level using CT images, and participants were categorized into quartiles based on the VSR. Logistic regression analyses were conducted to evaluate the association between VSR and the prevalence of gallstones, adjusting for age, body mass index (BMI), lifestyle factors, comorbidities, and biochemical markers.

**Results:**

The mean age and BMI were 52.3 ± 9.3 years and 24.6 ± 2.9 kg/m^2^, respectively. Gallstone prevalence increased progressively across VSR quartiles, from 4.0% in the lowest quartile to 7.6% in the highest (*p* < 0.001). In fully adjusted models, men in the highest VSR quartile had 1.6-fold higher odds of having gallstones compared to the lowest quartile (OR 1.596, 95% CI 1.074–2.373, *p* = 0.021).

**Conclusion:**

A higher VSR, reflecting a predominance of visceral over subcutaneous fat, was independently associated with an increased risk of gallstones in Korean men. These findings highlight the importance of abdominal fat distribution, beyond overall obesity, in gallstone pathogenesis. The VSR may serve as a valuable imaging biomarker for identifying men at elevated risk of developing gallstones.

## 1 Introduction

Gallstones are among the most common gastrointestinal disorders, with their incidence increasing globally due to changes in dietary habits, obesity, and metabolic health (1). The formation of gallstones is associated with various metabolic factors, including insulin resistance, dyslipidemia, and nonalcoholic fatty liver disease (NAFLD) (1). Obesity, in particular, has been consistently linked to an elevated risk of gallstones. However, recent studies suggest that not all patterns of fat distribution confer the same metabolic risk (2). Visceral fat, which envelops internal organs, is more metabolically active and proinflammatory than subcutaneous fat, and it significantly contributes to the development of metabolic disorders (3).

The visceral-to-subcutaneous fat ratio (VSR), ascertained through computed tomography (CT), has emerged as a valuable index reflecting the relative predominance of visceral fat over subcutaneous fat (4). In contrast to indirect or surrogate measures of visceral obesity, such as the visceral adiposity index, body mass index (BMI), or waist-to-hip ratio, which do not account for fat distribution, VSR offers a more precise assessment of metabolic risk by differentiating between harmful and benign adipose tissue compartments (4). An elevated VSR has been linked to insulin resistance, hepatic steatosis, and cardiovascular disease (5, 6). Nonetheless, it remains uncertain whether VSR is independently associated with the risk of gallstones, beyond traditional metabolic factors.

Previous research has predominantly examined the association between general obesity or visceral fat volume and gallstones, often without considering the balance between visceral and subcutaneous adiposity (7, 8). Given that subcutaneous fat may exert protective metabolic effects by buffering excess lipid storage, the ratio between these two adipose compartments may more accurately reflect metabolic health status. Consequently, elucidating the role of the VSR in gallstone formation could offer novel insights into the mechanisms connecting abdominal fat distribution and biliary disease.

Men and women demonstrate distinct patterns in abdominal fat distribution and gallstone pathophysiology. Men typically exhibit higher visceral fat accumulation relative to subcutaneous fat, whereas women generally have more subcutaneous fat (9). Estrogen-related effects in women increase the risk of cholesterol gallstones, resulting in sex-specific mechanisms of gallstone formation (10). Concentrating on men reduces heterogeneity and allows for a clearer assessment of the association between the VSR and gallstone risk.

Therefore, this study aimed to investigate the association between VSR and the presence of gallstones in a large cohort of Korean men.

## 2 Materials and methods

### 2.1 Study design and participants

**Figure 1:**
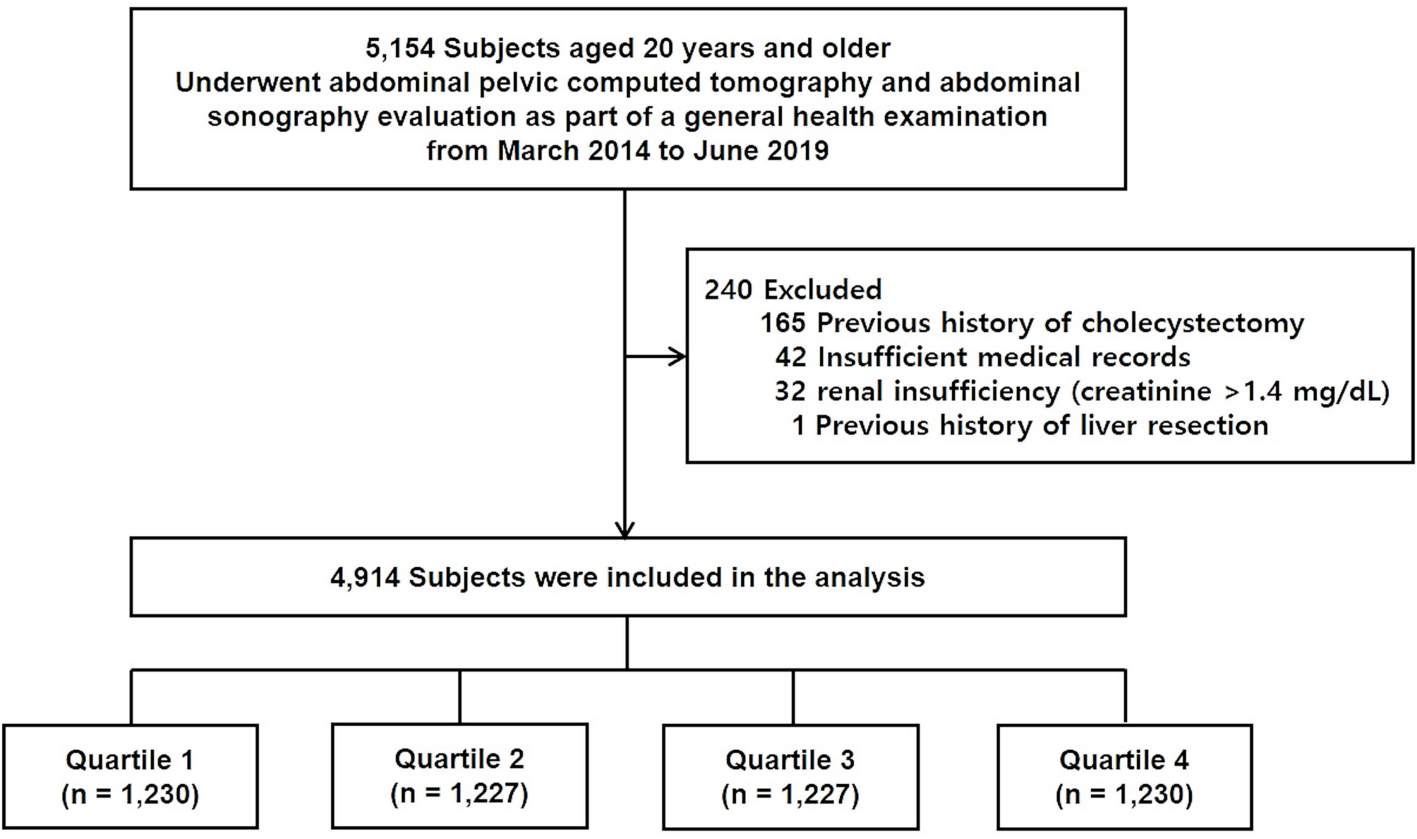
Overview of the study population.

In this retrospective cross-sectional study, 5,154 male participants initially took part, all of whom had both abdominopelvic computed tomography (CT) and abdominal ultrasonography during routine health examinations at a single tertiary medical center from March 2014 to June 2019. In Korea, abdominopelvic CT can be optionally included in comprehensive health screening programs at the request of individuals. These health checkup programs, which are widely available at tertiary hospitals and screening centers, are frequently utilized by asymptomatic adults for preventive assessment and are typically funded either by the individuals themselves or by their employers. Consequently, abdominopelvic CT may be conducted during routine health examinations even in the absence of specific clinical indications. Among 5,154 participants, subjects were excluded if they had previously undergone a cholecystectomy (*n* = 165), had incomplete or missing clinical data (*n* = 42), suffered from renal insufficiency (*n* = 32), or had a history of hepatic resection (*n* = 1). After these exclusion criteria were applied, the final analysis included 4,914 participants (Figure 1).

### 2.2 Clinical and laboratory measurements

Data from clinical and laboratory assessments were gathered from electronic health records and the institution’s clinical data warehouse. During routine health check-ups, standardized methods were employed to assess height, weight, and blood pressure (11). Blood samples, taken after fasting overnight, were evaluated for hematologic and biochemical markers. Hematologic assessments included counts of white blood cells, hemoglobin levels, and platelet counts. Biochemical evaluations involved measuring total bilirubin, albumin, aspartate aminotransferase (AST), and alanine aminotransferase (ALT). The lipid profile was composed of total cholesterol, low-density lipoprotein (LDL) cholesterol, high-density lipoprotein (HDL) cholesterol, and triglycerides. Other measured parameters included total calcium, fasting plasma glucose, hemoglobin A1c, and serum creatinine levels.

Diabetes mellitus was identified by a fasting plasma glucose level of ≥126 mg/dL, HbA1c of ≥6.5%, or a self-reported history of diabetes or the use of dietary changes or antidiabetic drugs. Hypertension was defined as having a systolic blood pressure of ≥140 mmHg, diastolic blood pressure of ≥90 mmHg, or a self-reported history of hypertension or the use of antihypertensive medications. Hyperlipidemia was characterized by a total cholesterol level of ≥240 mg/dL or a self-reported history of hyperlipidemia or the use of lipid-lowering drug (11).

### 2.3 Definition of alcohol use

Alcohol consumption was assessed using a self-administered questionnaire filled out during health examinations. Individuals who indicated that they drank alcoholic beverages at least once a week were identified as alcohol users, while those who drank less frequently or not at all were classified as non-users (12).

### 2.4 Definition of physical activity

To evaluate physical activity levels, a standardized questionnaire aligned with the World Health Organization (WHO) Global Recommendations on Physical Activity for Health (2010) (13) was utilized. Individuals classified as physically active if they participated in at least 150 min of moderate-intensity aerobic exercise or 75 min of vigorous-intensity exercise weekly, with each aerobic session lasting no less than 10 min.

### 2.5 Measurement of visceral fat and subcutaneous fat

Abdominppelvic CT scans were conducted using a SOMATOM Definition Flash system from Siemens Healthcare, located in Erlangen, Germany. To improve contrast, iopromide (Xenetix 350; Guerbet, Roissy, France) was administered intravenously at a concentration of 150 mg/mL. A total volume of 100–120 mL was delivered through an 18-gage cubital line at a speed of 3–4 mL/s, followed by a 20 mL saline flush at the same rate. Post-contrast images were captured 80 s after the initiation of the contrast injection. The CT acquisition protocol featured a beam collimation of 128 × 0.6 mm, a beam pitch of 0.6, a gantry rotation time of 0.5 s, and a field of view (FOV) tailored to the patient, with a tube voltage of 100 kVp. Radiation exposure was minimized using the CARE Dose 4D automatic exposure control system from Siemens Medical Solutions, Erlangen, Germany. Images were reconstructed with an I40f kernel and a slice thickness of 3 mm (14).

Body composition was assessed from CT images using Asan-J software, a modified version of ImageJ (NIH, Bethesda, MD, United States) (15). For each participant, two consecutive axial slices at the level of the inferior endplate of the L3 vertebra were chosen and averaged. Visceral and subcutaneous fat areas were measured using predefined adipose tissue thresholds (−190 to −30 Hounsfield units) in the software (Supplementary Figure 1) (14).

### 2.6 Identification of gallstones

Gallstones were identified through both abdominopelvic CT scans and abdominal ultrasound. In CT imaging, gallstones appeared as hyperdense spots within the gallbladder, characterized by well-circumscribed, high-attenuation areas that did not show contrast enhancement (16). The abdominal ultrasonography was conducted by experienced radiologists using high-resolution imaging equipment. To enhance the visibility of the biliary system, participants were instructed to fast for a minimum of 8 h before the procedure. On ultrasound, gallstones were diagnosed by their distinct features, such as echogenic spots with posterior acoustic shadowing and the movement of echoes when the patient’s position changed, indicating freely mobile intraluminal gallstones (16).

### 2.7 Statistical analysis

Participants were divided into four quartiles based on their VSR, as shown in Figure 1. Categorical variables are represented by frequencies and percentages, while continuous variables are shown as means with standard deviations. Group comparisons were conducted using Pearson’s chi-square test or Fisher’s exact test for categorical variables, and one-way ANOVA or the Kruskal–Wallis test for continuous variables, as appropriate.

Logistic regression analyses were utilized to assess the relationship between abdominal fat and gallstones. Both univariable and multivariable models were developed, with covariates for the multivariable analysis chosen based on clinical importance and statistical significance. Four progressive models were employed: Model 1 was unadjusted; Model 2 accounted for age and BMI; Model 3 included additional lifestyle and clinical factors such as alcohol use, physical activity status, hypertension, diabetes, dyslipidemia and fatty liver disease; and Model 4 further incorporated metabolic and biochemical markers like hemoglobin, albumin, total bilirubin, AST, ALT, triglycerides, HDL cholesterol, LDL cholesterol, fasting plasma glucose, hemoglobin A1c, and serum calcium. Odds ratios (ORs) with 95% confidence intervals (CIs) were calculated for all logistic regression models. A two-sided *p* value of less than 0.05 was deemed statistically significant. All statistical analyses were conducted using SPSS software (version 24; SPSS Inc., Chicago, IL, United States).

## 3 Results

The mean age of the 4,914 participants was 52.3 ± 9.3 years, and the mean BMI was 24.6 ± 2.9 kg/m^2^. The baseline characteristics of the study population according to quartiles of the VSR are summarized in Table 1. Participants in the highest quartile of VSR were significantly older and had higher BMI values compared with those in the lowest quartile. The prevalence of hypertension, hyperlipidemia, and fatty liver disease increased progressively across VSR quartiles (all *p* < 0.05). The proportion of current smokers also rose with increasing VSR, whereas the proportion of physically active individuals declined. No significant differences were observed in alcohol consumption across quartiles. In terms of laboratory parameters, participants with higher VSR values exhibited elevated levels of hemoglobin, AST, ALT, triglycerides, fasting plasma glucose, and hemoglobin A1c, along with lower HDL cholesterol levels (all *p* < 0.05). Total cholesterol showed a modest increasing trend, while LDL cholesterol and serum creatinine did not differ significantly among quartiles.

**Table 1:** Baseline characteristics of the study population according to the quartiles of the visceral-to-subcutaneous fat ratio.

Characteristics	Overall (*n* = 4,914)	Visceral fat to subcutaneous fat ratio				*p* value
		Quartile 1 < 0.6530 (*n* = 1,230)	Quartile 2 0.6531 ~ 0.9160 (*n* = 1,227)	Quartile 3 0.9160 ~ 1.2722 (*n* = 1,227)	Quartile 4 > 1.2723 (*n* = 1,230)	
Age, years	52.3 ± 9.3	48.3 ± 10.8	51.7 ± 8.7	53.7 ± 8.1	55.7 ± 7.8	< 0.001
BMI, kg/m^2^	24.6 ± 2.9	24.2 ± 3.4	24.4 ± 2.9	24.8 ± 2.7	25.1 ± 2.5	< 0.001
Diabetes mellitus	489 (10.0)	65 (5.3)	97 (7.9)	127 (10.4)	200 (16.3)	0.295
Hypertension	1,068 (21.7)	173 (14.1)	211 (17.2)	291 (23.7)	393 (32.0)	< 0.001
Hyperlipidemia	303 (6.2)	54 (4.4)	85 (6.9)	82 (6.7)	82 (6.7)	0.029
Fatty liver disease	1975 (40.2)	290 (23.6)	437 (35.6)	555 (45.2)	693 (56.3)	< 0.001
Current smoker	1,638 (33.9)	354 (29.3)	419 (34.9)	431 (35.5)	434 (36.0)	0.001
Alcohol user	3,957 (80.5)	967 (78.6)	991 (80.8)	994 (81.0)	1,005 (81.7)	0.243
Physically active	1,110 (22.6)	329 (26.7)	265 (21.6)	264 (21.5)	252 (20.5)	0.001
White blood cell, K/mmL	5.728 ± 1.7	5.454 ± 1.8	5.706 ± 1.6	5.831 ± 1.7	5.919 ± 1.7	0.072
Hemoglobin, g/dL	15.1 ± 1.1	15.0 ± 1.1	15.1 ± 1.1	15.2 ± 1.1	15.2 ± 1.2	0.020
Platelet, K/mmL	224.2 ± 49.2	222.1 ± 47.2	223.7 ± 48.0	225.7 ± 49.7	225.3 ± 51.8	0.304
Total bilirubin, mg/dL	0.967 ± 0.4	0.985 ± 0.4	0.974 ± 0.4	0.963 ± 0.4	0.945 ± 0.4	0.444
Albumin, g/dL	4.51 ± 0.3	4.54 ± 0.3	4.50 ± 0.3	4.50 ± 0.3	4.52 ± 0.3	0.954
AST, IU/L	26.3 ± 15.0	24.5 ± 11.2	25.9 ± 16.4	26.2 ± 13.9	28.7 ± 17.3	< 0.001
ALT, IU/L	30.7 ± 21.2	27.5 ± 19.7	29.9 ± 20.9	31.2 ± 18.9	34.3 ± 24.4	< 0.001
Total cholesterol, mg/dL	185.1 ± 38.1	182.2 ± 36.0	185.9 ± 37.6	186.4 ± 39.0	185.7 ± 39.6	0.011
LDL cholesterol, mg/dL	127.1 ± 35.3	124.6 ± 34.0	128.2 ± 35.4	128.3 ± 35.6	127.4 ± 36.2	0.060
HDL cholesterol, mg/dL	49.6 ± 13.8	53.1 ± 13.9	50.3 ± 13.7	48.5 ± 13.7	46.7 ± 13.2	0.037
Triglyceride, mg/dL	123.4 ± 80.5	99.4 ± 60.8	115.8 ± 70.3	131.1 ± 84.0	147.4 ± 94.6	< 0.001
Fasting blood glucose, mg/dL	97.7 ± 23.7	91.5 ± 18.1	96.0 ± 23.3	98.4 ± 23.5	104.8 ± 27.2	< 0.001
Hemoglobin A1c, %	5.67 ± 0.9	5.46 ± 0.7	5.63 ± 0.9	5.70 ± 0.8	5.88 ± 0.9	< 0.001
Serum creatinine, mg/dL	0.91 ± 0.1	0.90 ± 0.1	0.90 ± 0.1	0.91 ± 0.1	0.91 ± 0.1	0.653
Total calcium, mg/dL	9.26 ± 0.4	9.28 ± 0.4	9.24 ± 0.4	9.27 ± 0.4	9.28 ± 0.4	0.748

Values are shown as mean ± standard deviation or number (%). BMI, Body mass index; AST, Aspartate aminotransferase; ALT, Alanine aminotransferase; LDL, Low-density lipoprotein; HDL, High-density lipoprotein.

**Figure 2:**
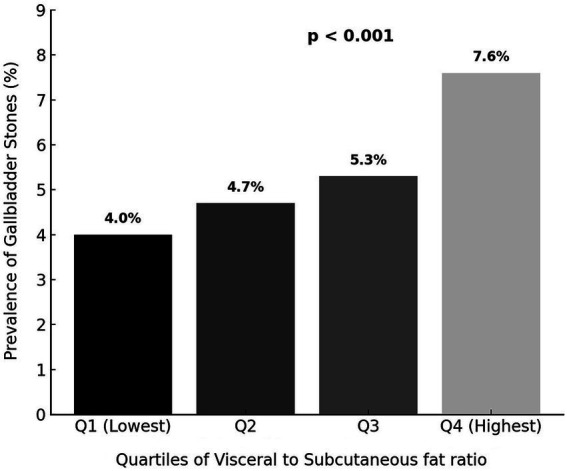
Prevalence of gallbladder stones according to quartiles of visceral-to-subcutaneous fat ratio.

Figure 2 illustrates the prevalence of gallstones according to VSR quartiles. The prevalence of gallstones increased steadily with higher VSR, rising from 4.0% in quartile 1 to 7.6% in quartile 4 (*p* < 0.001), indicating a positive relationship between greater visceral fat predominance and gallstone occurrence.

The associations between VSR quartiles and the risk of gallstones are presented in Table 2. In the unadjusted model (Model 1), participants in the highest quartile of VSR had nearly twice the odds of having gallstones compared with those in the lowest quartile (OR = 1.994, 95% CI 1.399–2.844, p < 0.001). This association remained significant even after sequential adjustment for potential confounders. In the fully adjusted model (Model 4), which accounted for demographic, lifestyle, clinical, and biochemical factors, the highest VSR quartile was independently associated with a greater risk of gallstones (OR = 1.596, 95% CI 1.074–2.373, *p* = 0.021).

**Table 2:** Association between abdominal fat and gallbladder stone according to the quartiles of the visceral-to-subcutaneous fat ratio.

Quartile of the visceral-to-subcutaneous fat ratio	Odds ratio (95% CI)	*p* value
Model 1		
Quartile 1 (reference)	1	
Quartile 2	1.196 (0.811–1.764)	0.367
Quartile 3	1.348 (0.922–1.971)	0.123
Quartile 4	1.994 (1.399–2.844)	< 0.001
Model 2		
Quartile 1 (reference)	1	
Quartile 2	1.101 (0.743–1.630)	0.631
Quartile 3	1.176 (0.797–1.734)	0.415
Quartile 4	1.646 (1.133–2.392)	0.009
Model 3		
Quartile 1 (reference)	1	
Quartile 2	1.117 (0.752–1.659)	0.583
Quartile 3	1.168 (0.787–1.734)	0.441
Quartile 4	1.574 (1.069–2.318)	0.022
Model 4		
Quartile 1 (reference)	1	
Quartile 2	1.130 (0.760–1.682)	0.546
Quartile 3	1.184 (0.794–1.767)	0.407
Quartile 4	1.596 (1.074–2.373)	0.021

CI = confidence interval. Model 1: Unadjusted. Model 2: Adjusted for age, body mass index. Model 3: Model 2 + alcohol use, physical activity status, hypertension, diabetes, dyslipidemia and fatty liver disease. Model 4: Model 3 + hemoglobin, albumin, total bilirubin, aspartate aminotransferase, alanine aminotransferase, triglyceride, high-density lipoprotein cholesterol, low-density lipoprotein cholesterol, fasting blood glucose, hemoglobin A1c, and serum calcium.

## 4 Discussion

This large-scale cross-sectional study investigated the relationship between the VSR and the presence of gallstones in Korean men. The results indicated that a higher VSR was notably linked to a greater prevalence of gallstones, even after accounting for age, BMI, and various metabolic and biochemical factors. Participants in the highest quartile of VSR had approximately 1.6-fold higher odds of having gallstones compared with those in the lowest quartile. These findings suggest that an imbalance favoring visceral over subcutaneous fat may contribute to gallstone formation independently of general obesity.

The present findings align with previous evidence linking visceral adiposity to a wide spectrum of metabolic and hepatobiliary disorders (2, 7). Visceral fat is metabolically more active and proinflammatory than subcutaneous fat, releasing free fatty acids, adipokines, and cytokines that contribute to insulin resistance, hepatic steatosis, and dyslipidemia (3). These metabolic disturbances may play a pivotal role in biliary cholesterol supersaturation and gallstone formation. Several studies have also demonstrated that the VSR serves as a more sensitive and integrated indicator of metabolic risk compared to traditional anthropometric indices such as BMI or waist circumference, because it captures the relative predominance of metabolically detrimental visceral fat over metabolically protective subcutaneous fat (4, 17). Furthermore, higher VSR values have been closely associated with insulin resistance, hepatic lipid deposition, and systemic inflammation (5, 6), all of which are crucial intermediates in the development of gallstone disease. Collectively, our results support the concept that an elevated VSR reflects an adverse metabolic milieu that predisposes individuals to gallstone pathogenesis.

Previous studies examining the association between obesity and gallstone formation have predominantly focused on overall or visceral fat accumulation, often overlooking the balance between visceral and subcutaneous fat compartments (7, 8). In contrast to visceral fat, subcutaneous fat may play a protective or buffering role by safely storing excess lipids and alleviating metabolic stress (18), thereby influencing overall metabolic resilience. Traditional assessments that consider only absolute visceral fat volume may fail to capture this critical interplay between fat depots. By integrating both visceral and subcutaneous fat measurements into a single index, the VSR, our study provides a more nuanced evaluation of abdominal adiposity. We demonstrate that the relative predominance of visceral fat, rather than the absolute quantity alone, serves as a stronger predictor of gallstone risk. This distinction is significant, as two individuals with the same visceral fat area may differ substantially in their metabolic health depending on their subcutaneous fat reserves.

Several biological mechanisms may elucidate the observed association between elevated VSR and gallstone formation. Visceral adipose tissue promotes systemic inflammation through the secretion of tumor necrosis factor-*α*, interleukin-6, and other proinflammatory cytokines, which subsequently lead to insulin resistance and altered hepatic lipid metabolism (19, 20). Insulin resistance enhances hepatic cholesterol synthesis and biliary cholesterol saturation, both of which are critical steps in the formation of cholesterol gallstones (21). Moreover, the accumulation of visceral fat has been associated with reduced gallbladder motility, thereby promoting bile stasis and crystallization (22). Additionally, increased oxidative stress and altered bile acid metabolism in visceral obesity may further contribute to the pathogenesis of gallstones (23, 24). In contrast, subcutaneous fat functions as a metabolic reservoir that mitigates lipotoxicity by safely storing excess lipids (17, 18). Consequently, a higher VSR, indicative of visceral fat predominance, may represent a state of metabolic imbalance that predisposes individuals to the development of gallstones.

Our findings also have potential clinical implications. The VSR, as measured using CT scans, could serve as a practical and reliable imaging biomarker for evaluating the risk of gallstone formation, particularly in populations undergoing abdominal CT for routine health examinations or other medical purposes. Unlike BMI, which does not distinguish between visceral and subcutaneous fat compartments, VSR offers a more precise representation of abdominal fat distribution and its metabolic consequences. By identifying individuals with a higher relative proportion of visceral fat, clinicians may more effectively detect those at increased risk for gallstones and related metabolic disturbances. Incorporating VSR assessment into standard health check-up protocols could enhance early recognition of metabolically vulnerable individuals, providing additional prognostic information that complements conventional anthropometric indices. Future research should investigate whether VSR-guided risk stratification can inform personalized lifestyle interventions, dietary modifications, or other preventive strategies aimed at reducing visceral fat accumulation and ultimately lowering the incidence of gallstone disease.

The exclusive inclusion of male participants in this study distinguishes it from numerous prior epidemiological investigations on gallbladder stones, which predominantly focused on female or mixed-gender populations (25, 26). This focus is pertinent due to the significant sex-based differences in fat distribution and the biological mechanisms underlying gallstone formation. Women generally accumulate more subcutaneous fat and are predisposed to cholesterol gallstones, primarily due to estrogen-induced increases in hepatic cholesterol secretion and bile saturation (27). In contrast, men typically exhibit a higher proportion of visceral adiposity, even at comparable BMI levels (28), which promotes insulin resistance, hepatic lipid accumulation, and dyslipidemia - processes closely associated with gallstone development. The VSR provides a useful composite indicator of the relative dominance of visceral over subcutaneous fat, thereby reflecting metabolic imbalance more accurately than absolute fat volume alone. Our findings indicate that in men, a higher VSR may serve as a marker of increased metabolic susceptibility contributing to gallstone risk. These observations highlight the potential influence of sex hormones and adipose tissue distribution on gallstone pathophysiology and suggest that future studies including both sexes are warranted to clarify whether the predictive value of VSR differs across sex and hormonal profiles.

Several limitations of this study should be acknowledged. First, due to its cross-sectional design, causal relationships between VSR and gallstone formation cannot be established. Second, our study population consisted exclusively of men from a single tertiary medical center, which may limit the generalizability of the findings to women, other ethnicities, or patients with symptomatic gallstone disease. Third, despite adjustments for various demographic, lifestyle, and biochemical factors, the possibility of residual confounding from unmeasured variables, such as detailed dietary habits, family history of gallstones, or genetic predisposition, cannot be entirely dismissed. In addition, although waist circumference is commonly used to assess abdominal adiposity, it cannot distinguish visceral from subcutaneous fat. We prioritized CT-derived measurements for precise fat distribution assessment and adjusted for overall body size using BMI, which we believe adequately accounts for abdominal adiposity. Finally, although CT-based body composition analysis offers precise quantification of visceral and subcutaneous fat, its application involves radiation exposure and higher costs, which may constrain its feasibility for large-scale population screening or routine clinical use.

## 5 Conclusion

In conclusion, a higher VSR was significantly associated with an increased risk of gallstones in Korean men, independent of BMI and other metabolic factors. These results emphasize that abdominal fat distribution, particularly the relative predominance of visceral over subcutaneous adiposity, plays an important role in gallstone pathogenesis. The VSR may serve as a valuable imaging biomarker for identifying individuals at increased risk of developing gallstones.

## Data Availability

The datasets presented in this article are not readily available because the datasets used and/or analyzed the current study are available from the corresponding author on reasonable request. Requests to access the datasets should be directed to Doo-Ho Lim, dlaengh@hanmail.net.
