# Supplementary figures and images for: Visceral-to-subcutaneous fat ratio and risk of gallstones in Korean men: an observational study of 4,914 cases
Source: Front Med (Lausanne). 2025 Dec 8;12:1720552. doi: 10.3389/fmed.2025.1720552 (PMC12719422; doi:10.3389/fmed.2025.1720552)

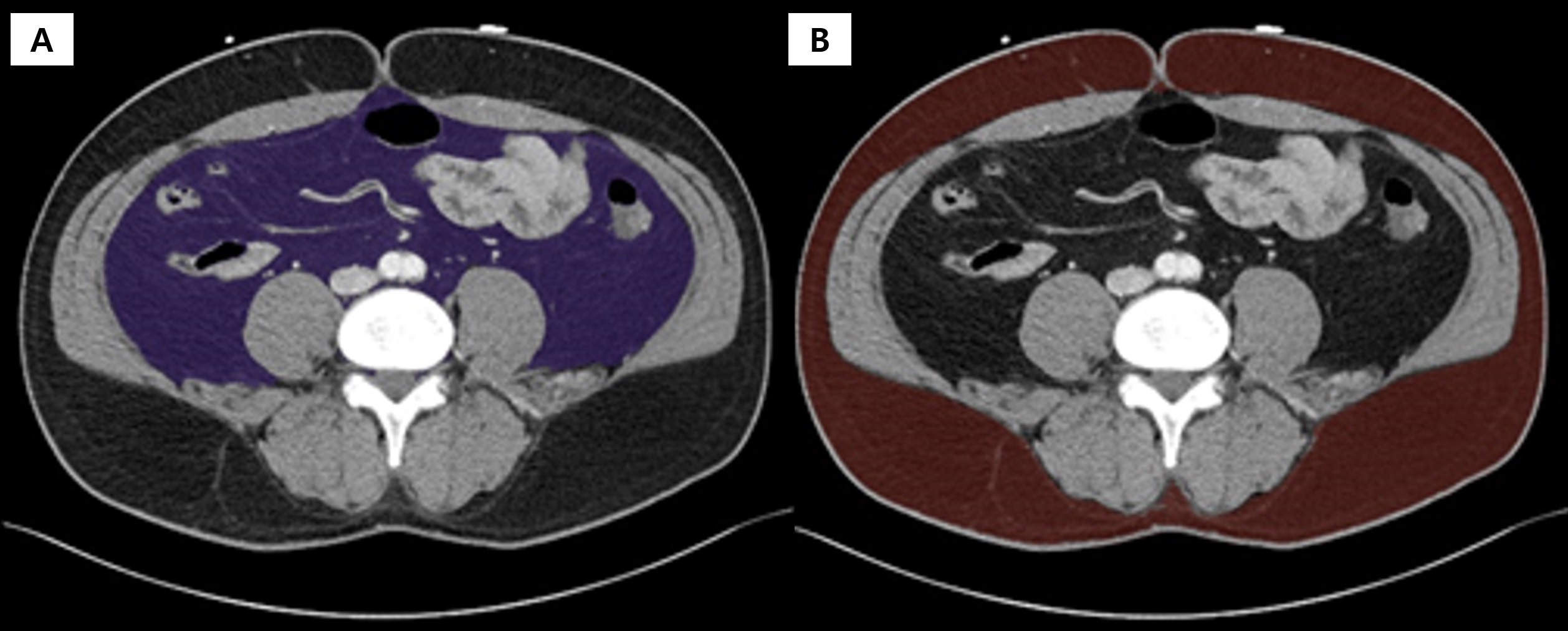

Supplement: Supplementary file 1 [file Image_1.TIF]
